# Severely Heat Injured Survivors of *E. coli* O157:H7 ATCC 43888 Display Variable and Heterogeneous Stress Resistance Behavior

**DOI:** 10.3389/fmicb.2016.01845

**Published:** 2016-11-18

**Authors:** Elisa Gayán, Sander K. Govers, Chris W. Michiels, Abram Aertsen

**Affiliations:** Laboratory of Food Microbiology, Department of Microbial and Molecular Systems, Faculty of Bioscience Engineering, KU LeuvenLeuven, Belgium

**Keywords:** *E. coli* O157:H7, sublethal injury, resuscitation, heat resistance, high hydrostatic pressure resistance

## Abstract

Although minimal food processing strategies aim to eliminate foodborne pathogens and spoilage microorganisms through a combination of mild preservation techniques, little is actually known on the resistance behavior of the small fraction of microorganisms surviving an inimical treatment. In this study, the conduct of severely heat stressed survivors of *E. coli* O157:H7 ATCC 43888, as an indicator for the low infectious dose foodborne enterohemorrhagic strains, was examined throughout their resuscitation and outgrowth. Despite the fact that these survivors were initially sublethally injured, they were only marginally more sensitive to a subsequent heat treatment and actually much more resistant to a subsequent high hydrostatic pressure (HHP) shock in comparison with unstressed control cells. Throughout further resuscitation, however, their initial HHP resistance rapidly faded out, while their heat resistance increased and surpassed the initial heat resistance of unstressed control cells. Results also indicated that the population eventually emerging from the severely heat stressed survivors heterogeneously consisted of both growing and non-growing cells. Together, these observations provide deeper insights into the particular behavior and heterogeneity of stressed foodborne pathogens in the context of food preservation.

## Introduction

Consumer demand for fresh-like foods challenges the food industry to design minimal processing strategies that improve the retention of the nutritional and sensorial properties of the food while ensuring its microbial safety and stability. In this context, the intelligent combination of mild preservation factors (or hurdles) that can act additively or even synergistically to inhibit or inactivate foodborne pathogens and spoilage microorganisms presents an interesting venue (Pasha et al., 2014). In fact, different potential hurdles exist, ranging from temperature, pH, water activity, antimicrobial compounds to emerging technologies such as high hydrostatic pressure (HHP) and pulsed electric fields (PEF) (Raso and Barbosa-Canovas, 2003; Pasha et al., 2014), and a number of potent combinations among them have already been observed. As such, it was found that the recovery and viability of cells surviving HHP or PEF treatment becomes compromised during storage in acidified products (Garcia-Graells et al., 1998; García et al., 2003), while the simultaneous combination of mild heat with HHP or PEF has proven to synergistically inactivate pathogenic bacteria (Patterson and Kilpatrick, 1998; Amiali et al., 2007; Torres et al., 2016). Likewise, some natural antimicrobial compounds (e.g., essential oils, reuterin, nisin) or enzymes (e.g., lysozyme, lactoperoxidase) have been shown to act synergistically with mild heat, PEF, or HHP in inactivating pathogens, thereby allowing a reduction in the intensity of the physical treatment and the concentration of antimicrobials and consequent impact on food quality (Masschalck et al., 2001; Saldaña et al., 2012; Ait-Ouazzou et al., 2013; Feyaerts et al., 2015; Montiel et al., 2015).

However, while these examples underscore the potential of hurdle technology, a successful combination of hurdles is often made serendipitously, with little or no insights in the mechanisms underlying the possible synergy or antagonism between different hurdles in terms of microbial inactivation (i.e., when the lethal effect of the combined treatment is higher or lower, respectively, than the sum of the effect of each individual hurdle). Indeed, despite the multitude of available hurdles, their bacteriostatic or bactericidal mode of action often remains cryptic or difficult to study because of the multitarget impact on the cell. Furthermore, little is known about the physiological response and subsequent behavior that is triggered in the small fraction of cells that manage to survive one or more mild hurdles, often because of the technical difficulties in properly studying the features or dynamics specifically occurring within a minute subpopulation.

In order to be able to proceed toward a more purposeful design of hurdle approaches, a better understanding of the physiology and behavior of severely stressed but sublethally injured survivors (i.e., cells that are alive but nevertheless unable to survive or proliferate in media containing selective agents; Wu, 2008) in terms of their potential stress response and resistance development is of great importance. In fact, when considering the conduct of severely stressed cells, different possible scenarios with opposing outcomes can be envisaged. For example, it could be anticipated that the small numbers of cells surviving a severe stress inevitably incur a debilitating extent of injury that renders them hypersensitive to any subsequent stress exposure. Alternatively, however, it could be that the individual cellular stress resistance is a variable trait within an isogenic population, and that the survivors of a stressful encounter therefore tend to become enriched in a phenotypically resistant subfraction of the original population, which could be better equipped to deal with a subsequent stress (Wesche et al., 2009). Moreover, both scenarios might arise within the same population of survivors as well.

Since such survival dynamics can be of particular importance for low infectious dose foodborne pathogens such as Enterohemorrhagic *Escherichia coli* (EHEC), this study set out to scrutinize and compare the resistance behavior of unstressed and severely heat stressed populations of *E. coli* O157:H7 (strain ATCC 43888).

## Materials and Methods

### Bacterial Strains and Growth Conditions

*Escherichia coli* O157:H7 strain ATCC^®^ 43888^TM^, a human fecal isolate obtained from the American Type Culture Collection, was used throughout this study. This strain lacks the genes for Shiga toxins I and II, and was chosen as a laboratory surrogate for EHEC O157:H7 strains because of its attenuated virulence. Stationary phase cultures were obtained by inoculating test tubes containing 4 ml of Tryptone Soy Broth (TSB; Oxoid, Basingstoke, UK) with a single colony grown on a Tryptone Soy Agar (TSA) plate, and then incubated aerobically with shaking (300 rpm) for 18 h at 37°C.

### Heat and HHP Treatment

Cells were harvested by centrifugation (4000 × *g*, 5 min) and resuspended in an equal volume of a physiological concentration of 0.85% KCl (Sigma-Aldrich, St. Louis, MO, USA). When the impact of translational capacity was assayed, a final concentration of 10 μg/ml of chloramphenicol (Sigma-Aldrich) was added to this suspension. For thermal treatment, three sterile PCR tubes were each aseptically filled with 75 μl of resuspended cells and subjected to heat (53, 56, or 80°C) for 15 min using a PCR apparatus (Tpersonal 48, Biometra GmbH, Goettingen, Germany). For HHP treatment, on the other hand, 200 μl of the suspension was heat sealed in a sterile polyethylene bag after exclusion of the air bubbles and subjected to pressure (300, 400, and 500 MPa) for 15 min in an 8-ml pressure vessel (HPIU-10000, 95/1994; Resato, Roden, The Netherlands), held at 20°C with an external water jacket connected to a cryostat. Please note that both the slow pressure increase (100 MPa/min) and the external water jacket attenuate adiabatic heating during pressure build-up. Finally, decompression was almost instantaneous. After heat or HHP treatment, samples were aseptically retrieved from the PCR tubes or polyethylene bags, respectively, and survival was determined as described below.

### Determination of Viability

Samples were serially diluted in 0.85% KCl supplemented with 0.1% bacteriological peptone (Oxoid), and subsequently spot-plated (5 μl) or spread-plated (100 μl) on TSA. Thus, the detection limit was 200 or 10 CFU/ml for spot- or spread-plating samples, respectively. Where indicated, cells were also recovered in Violet Red Bile Glucose Agar (VRBGA; Oxoid) in order to determine the extent of sublethal injury. After 24 h of incubation at 37°C, the colonies were counted and the logarithmic number of colony forming units per ml (CFU/ml) prior (log_10_ *N*_0_) and after (log_10_ *N*) treatment was calculated. The logarithmic reduction factor was calculated as log_10_ (*N*_0_/*N*).

### Heat and HHP Resistance of Unstressed or Heat Stressed Populations

Heat stressed populations were prepared by subjecting stationary phase cells of *E. coli* ATCC 43888 to a heat treatment of 56°C (15 min) in 0.85% KCl (supplemented with 10 μg/ml of chloramphenicol when the impact of translational capacity was assayed). The corresponding control population (without previous exposure to thermal stress) was prepared by diluting the stationary phase culture 1/10,000 in the treatment medium to eventually reach the same concentration of viable cells than in heat treated samples (see Section “Preparation of Unstressed and Heat Stressed Populations of *E. coli* O157:H7 ATCC 43888”). Subsequently, an aliquot of the treated and control sample was diluted 1/10 into fresh, prewarmed TSB (supplemented with 10 μg/ml of chloramphenicol when the impact of translational capacity was assayed), which was previously supplemented with a concentration of 10^8^ death cells/ml obtained by exposure of stationary phase cells to a heat shock of 80°C (15 min) in 0.85% KCl.

Subsequently, the obtained stressed and control populations were incubated with shaking (300 rpm) at 37°C. An aliquot of each population was regularly taken in 1 h intervals and resuspended in an equal volume of 0.85% KCl (supplemented with 10 μg/ml of chloramphenicol when the impact of translational capacity was assayed) to determine viability and resistance to heat (53°C, 15 min) and HHP (300 MPa, 15 min). When indicated, a final concentration of 100 μg/ml of ampicillin (Fischer Scientific, Pittsburgh, PA, USA) was added to both control and heat stressed populations at the specified incubation time.

### Checking for Heat or HHP Resistant Mutants Among Survivors

In order to check whether a population of survivors was mainly composed of wild-type cells or was vastly enriched in heat or HHP resistant mutants, 10–20 randomly picked colonies of the surviving population recovered on TSA were regrown in TSB to stationary phase (37°C, 18 h) and challenged to heat (56°C, 15 min) or HHP shock (300 MPa, 15 min). Clones not displaying a statistically significantly higher (*P* ≤ 0.05) heat or HHP resistance than the parental strain were considered to be wild-type.

### Statistical Analysis

Statistical analyses, ANOVA and *t*-test, were carried out using the software GraphPad PRISM 5.0 (GraphPad Software Inc., San Diego, CA, USA), and differences were regarded as significant when *P* was ≤0.05. All microbial inactivation outcomes shown in figures correspond to averages and standard deviations calculated from three replicates.

## Results

### Preparation of Unstressed and Heat Stressed Populations of *E. coli* O157:H7 ATCC 43888

**FIGURE 1:**
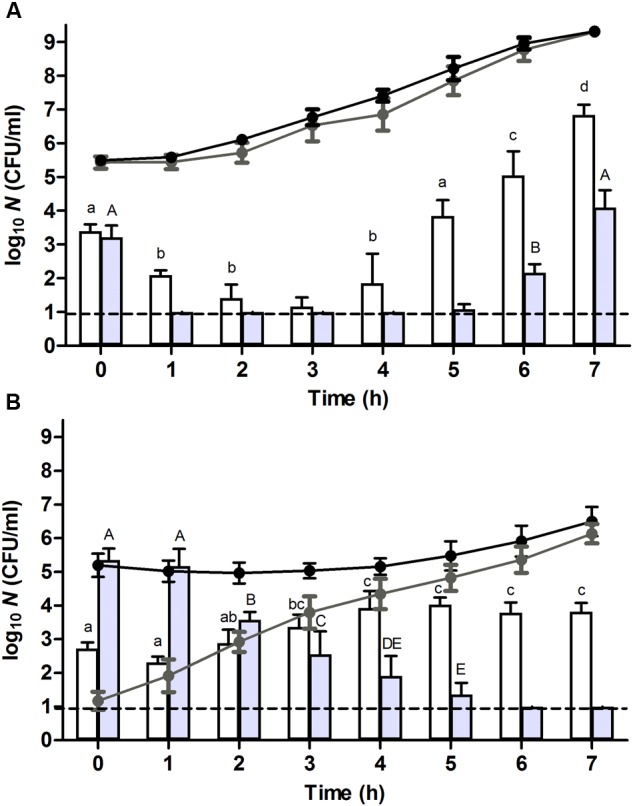
**Resuscitation and heat or high hydrostatic pressure (HHP) resistance of (A)** unstressed control cells or **(B)** heat stressed (56°C, 15 min, in 0.85% KCl) survivors of an *E. coli* ATCC 43888 population during incubation in tryptone soy broth (TSB) at indicated time points. Black and gray line graphs show the evolution of viability of the corresponding populations on tryptone soy agar (TSA) and violet red bile glucose agar (VRBGA), respectively, during incubation at 37°C. White and gray bars represent the number of survivors determined on TSA of the corresponding populations subjected to a heat (53°C, 15 min) or HHP shock (300 MPa, 15 min), respectively, in 0.85% KCl. Viability is expressed as log_10_(CFU/ml) on the indicated plating medium, and the dotted line represents the detection limit of 10 CFU/ml. Lowercase and capital letters indicate statistically significant differences (*P* ≤ 0.05) in the survival to the 53°C heat or 300 MPa HHP shock, respectively, among different sampling times.

Heat stressed populations were generated by exposing a stationary phase population of *E. coli* ATCC 43888 (after resuspension in 0.85% KCl) to 56°C for 15 min, thereby causing a ca. 3.2 log_10_ cycle reduction in viability (expressed as CFU/ml on TSA). Since this inevitably implied that the resuscitating survivors that form the further subject of this study are surrounded by a majority of heat inactivated siblings, an appropriate control population was prepared by diluting an unstressed stationary phase population of ATCC 43888 1/1,000 in 0.85% KCl, after which both control and heat stressed preparations were additionally diluted 1/10 in fresh TSB medium containing 10^8^ CFU/ml of completely heat inactivated (80°C, 15 min) stationary phase ATCC 43888 cells. This finally resulted in a population of (i) ca. 10^5^ CFU/ml of heat stressed (56°C, 15 min) survivors, or (ii) ca. 10^5^ CFU/ml of unstressed control cells, both similarly surrounded by a majority of heat inactivated siblings (**Figure 1**).

In order to examine whether the heat stressed survivors were enriched in mutants displaying increased heat resistance, 20 random individual colonies emerging from the heat stressed survivors were regrown to stationary phase and once more subjected to a heat shock of the same magnitude (i.e., 56°C, 15 min). This revealed that their resistance was indistinguishable from that of the parental population (data not shown), indicating that the majority of heat stressed survivors were not composed of heat resistant mutants.

### Growth and Resuscitation Dynamics of Unstressed and Heat Stressed Populations of *E. coli* O157:H7 ATCC 43888

Upon further incubation of these populations at 37°C, the plate counts on TSA indicated that control cells displayed virtually no lag time and committed to growth already after 1 h (**Figure 1A**, black line), while the growth of heat stressed survivors only became evident ca. 5 h after the 56°C heat shock (**Figure 1B**, black line). In addition, counts on selective VRBGA plates indicated that control cells displayed no signs of sublethal injury (**Figure 1A**, gray line), while directly after the 56°C heat shock the vast majority (i.e., >99.99%) of heat stressed survivors was sublethally injured (**Figure 1B**, gray line). Once resuscitation proceeded, counts of the stressed survivors on VRBGA plates steadily increased (**Figure 1B**, gray line).

**FIGURE 2:**
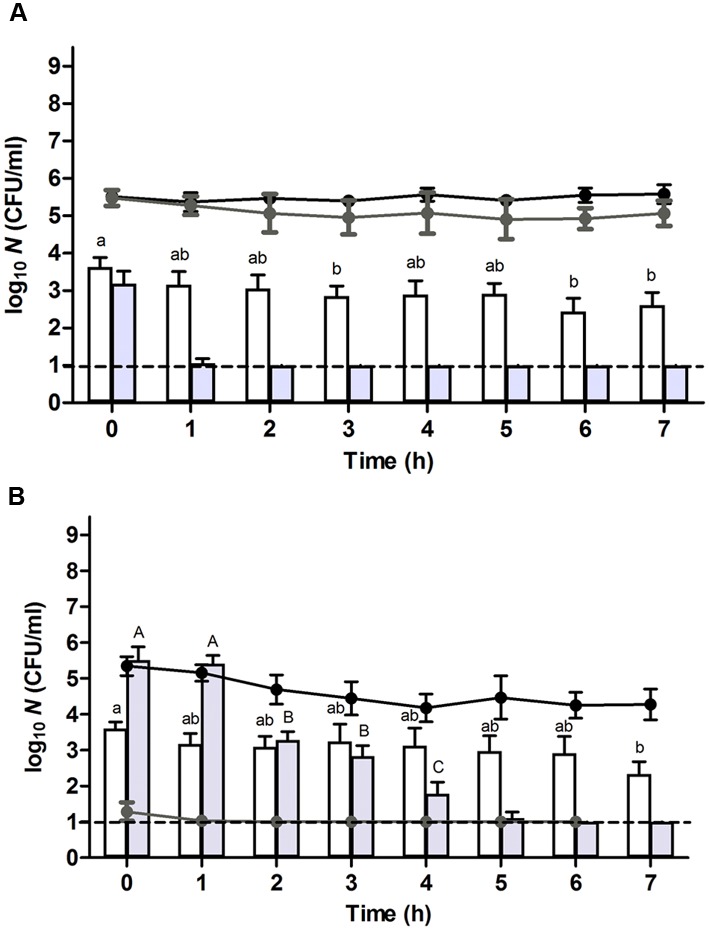
**Resuscitation and heat or HHP resistance of (A)** unstressed control cells or **(B)** heat stressed (56°C, 15 min, in 0.85% KCl supplemented with 10 μg/ml of chloramphenicol) survivors of an *E. coli* ATCC 43888 population during incubation in chloramphenicol (10 μg/ml) supplemented TSB at indicated time points. Black and gray line graphs show the evolution of viability of the corresponding populations on TSA and VRBGA, respectively, during incubation at 37°C. White and gray bars represent the number of survivors determined on TSA of the corresponding populations subjected to a heat (53°C, 15 min) or HHP shock (300 MPa, 15 min), respectively, in 0.85% KCl in the presence of chloramphenicol (10 μg/ml). Viability is expressed as log_10_(CFU/ml) on the indicated plating medium, and the dotted line represents the detection limit of 10 CFU/ml. Lowercase and capital letters indicate statistically significant differences (*P* ≤ 0.05) in the survival to the 53°C heat or 300 MPa HHP shock, respectively, among different sampling times.

In order to evaluate the importance of translational capacity on the process of resuscitation, control and heat stressed populations were prepared and incubated as described above, but this time in the continuous presence of chloramphenicol, an antibiotic that inhibits the translational apparatus of the cell (Pestka, 1974). Consistent with the bacteriostatic nature of chloramphenicol, growth of the control cells ceased while their viability remained unaffected (**Figure 2A**, black line). Moreover, only a marginal effect could be noted on the sublethal injury of the control cells (**Figure 2A**, gray line). In contrast, the presence of chloramphenicol compromised the recovery of the heat stressed survivors on VRBGA plates (**Figure 2B**, gray line), indicating that translational capacity is a prerequisite for proper resuscitation. Furthermore, likely as a result of this compromised resuscitation, the viability of the heat stressed survivors dropped ca. 1 log over the next 3–4 h, after which it remained constant (**Figure 2B**, black line).

### Evolution of Heat Resistance during Resuscitation and Growth of Unstressed and Heat Stressed Populations of *E. coli* O157:H7 ATCC 43888

In order to compare the behavior of unstressed control cells and their severely heat stressed counterparts in terms of stress resistance, both populations were sampled at regular time intervals during their resuscitation and/or growth, and examined for their resistance to a heat shock of 53°C for 15 min after resuspension in 0.85% KCl. This revealed that the initial heat resistance of the previously heat stressed survivors (i.e., reduction factor of ca. 2.5 log_10_ cycles at *t* = 0 h; **Figure 1B**, white bars) was only slightly lower than that of control cells (i.e., reduction factor of ca. 2.1 log_10_ cycles at *t* = 0 h; **Figure 1A**, white bars), despite the fact that the former population was sublethally injured. Importantly, the population of heat stressed survivors that survived the second (53°C) heat shock was not composed of heat resistant mutants, since 10 of their randomly picked and regrown individual colonies did not display an increased heat resistance (after a 56°C shock for 15 min) compared to the parental strain (data not shown).

Throughout further incubation, the initial heat resistance of control cells drastically declined over the next 3 h, after which it gradually started to increase again (**Figure 1A**, white bars), likely correlating with cells respectively passing through the exponential growth phase (from *t* = 1–5 h; during which they are at their most sensitive state) and subsequently entering the stationary growth phase [at *t* = 5–7 h; during which general stress resistance emerges (Ihssen and Egli, 2004; Berney et al., 2006)]. In fact, the presence of chloramphenicol could counteract this initial loss of heat resistance (**Figure 2A**, white bars), likely by preventing these cells to enter the exponential phase of growth and thus enabling them to retain stationary phase resistance. In contrast, throughout the ca. 4 h resuscitation phase the population of heat stressed survivors did not uniformly commit to growth and incurred an increasing heat resistance up to the point where the second heat shock only managed to inactivate ca. 1.1 log_10_ cycles (at *t* = 4 h) (**Figure 1B**, white bars), which is even 10-fold less than the inactivation achieved with the unstressed control population at *t* = 0 h.

Interestingly, while after 4 h the population stemming from the heat stressed survivors displayed increasing TSA counts (**Figure 1B**, black line), the absolute number of cells surviving the second (53°C) heat shock (**Figure 1B**, white bars) appeared to remain unaltered. This contrasted with the readily declining number of (53°C) heat shock survivors in exponentially growing control cells (**Figure 1A**, white bars), and suggested that a fraction of the cells within the population stemming from the stressed survivors had not entered the exponential phase of growth. Moreover, in the presence of chloramphenicol, the absolute number of cells surviving the second (53°C) heat shock remained relatively constant during the first 4 h (**Figure 2B**, white bars), although the total number of viable cells meanwhile dropped ca. 1 log (**Figure 2B**, black line). If the mounting of a stress response would be completely inhibited by the chloramphenicol, this observation could suggest the presence of a residual subfraction within the heat stressed survivors with a phenotypically increased heat resistance.

**FIGURE 3:**
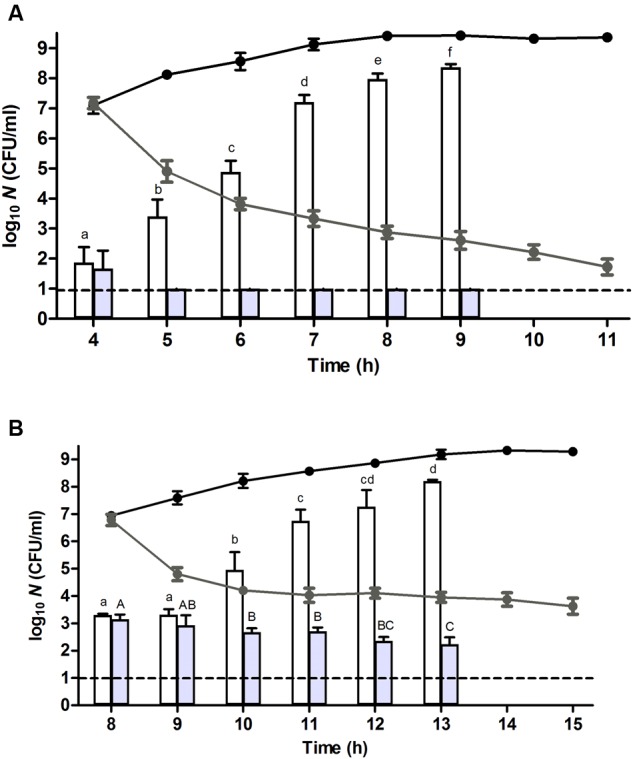
**Resuscitation, heat resistance, and ampicillin sensitivity of a (A)** unstressed control cells and **(B)** heat stressed (56°C, 15 min, in 0.85% KCl) survivors of an *E. coli* ATCC 43888 population after reaching ca. 10^7^ CFU/ml (i.e., at *t* = 4 and 8 h for control and heat treated population, respectively) during incubation in TSB (37°C) at indicated time points. Black and gray line graphs represent the evolution of viability on TSA of the corresponding populations in the absence or presence of ampicillin (100 μg/ml), respectively. White and gray bars represent the number of survivors determined on TSA of the corresponding populations in the absence or presence of ampicillin (100 μg/ml), respectively, subjected to a heat shock (53°C, 15 min) in 0.85% KCl. Viability is expressed as log_10_(CFU/ml) on the indicated plating medium, and the dotted line represents the detection limit of 10 CFU/ml. Lowercase and capital letters indicate statistically significant differences (*P* ≤ 0.05) in the survival of the corresponding populations in the absence or presence of ampicillin, respectively, to the second heat shock (53°C) among different sampling times.

To investigate this issue of heterogeneity more closely, the growth displaying populations stemming (i) from the control cells after 4 h of incubation, and (ii) from the heat stressed survivors after 8 h of incubation (both harboring ca. 10^7^ CFU/ml) were exposed to ampicillin, an antibiotic that is lethal to growing cells (Balaban et al., 2004). Interestingly, while the control population steadily became inactivated ca. 5.5 log_10_ cycles over the subsequent 7 h (at *t* = 11 h; **Figure 3A**, gray line), the heat stressed population still harbored a ca. 4 × 10^3^ CFU/ml fraction of ampicillin resistant cells (at *t* = 15 h; **Figure 3B**, gray line). This observation indicated that a considerable fraction of the heat stressed survivors did not readily commit to growth even though the population as a whole increased in numbers. Consequently, it seemed that the original population of heat stressed survivors gradually became split into (i) an exponentially growing and consequently heat sensitive fraction that likely originated from early resuscitated cells and that enriched in the population to become dominant (and detectable on TSA) after ca. 4 h, and (ii) a non-growing (ampicillin tolerant) fraction that retained its original or acquired heat resistance (**Figure 3B**, gray lines and gray bars). Please note that in the absence of ampicillin the growing fraction would eventually reach stationary phase and concomitantly become resistant as well (**Figure 3B**, black lines and white bars).

### Evolution of High Hydrostatic Pressure Resistance during Resuscitation and Growth of Unstressed and Heat Stressed Populations of *E. coli* O157:H7 ATCC 43888

**FIGURE 4:**
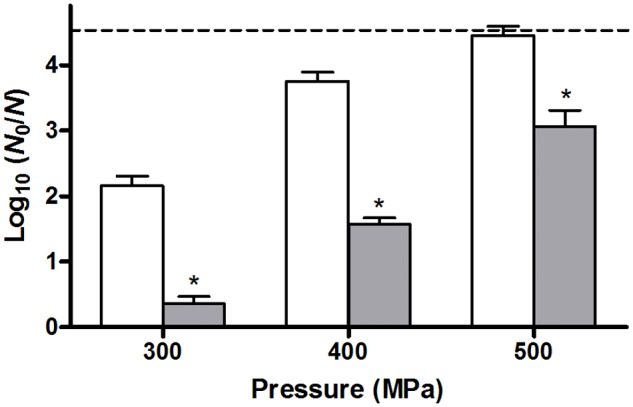
**Inactivation of unstressed control cells (white bars) or heat stressed (56°C, 15 min) survivors (gray bars) of an *E. coli* ATCC 43888 population after a HHP shock of 300, 400, or 500 MPa (15 min) in 0.85 % KCl.** Inactivation is expressed as log_10_(*N*_0_/*N*) on TSA plating medium, and the dotted line represents the detection limit of 10 CFU/ml. Asterisk indicates statistically significant differences (*P* ≤ 0.05) between the HHP inactivation of heat shocked cells and control cells for each pressure treatment.

In order to also compare the behavior of unstressed control cells and their heat stressed counterparts in terms of resistance against another (non-thermal) stressor such as HHP, both populations were sampled at regular time intervals during their resuscitation and/or growth, and examined for their resistance to a HHP shock of 300 MPa for 15 min after resuspension in 0.85% KCl. Surprisingly, while directly after the preparation of the samples (i.e., *t* = 0 h; **Figure 1A**, gray bars) control cells suffered ca. 2.3 log_10_ cycles of inactivation, the heat stressed sublethally injured survivors appeared to be completely unharmed by the HHP shock (**Figure 1B**, gray bars). However, also here it could be confirmed that the population of heat stressed survivors that survived the subsequent HHP shock was not composed of HHP resistant mutants, since ten of their randomly picked and regrown individual colonies did not display an increased HHP resistance (after a 300 MPa shock for 15 min) compared to the parental strain (data not shown). This heat induced HHP resistance could even be observed at challenge pressures of 400 and 500 MPa as well, although these more intense HHP exposures also progressively decreased the survival of the heat stressed cells (**Figure 4**).

Throughout further incubation, and similar to the evolution of their heat resistance, the initial 300 MPa resistance displayed by the unstressed control cells rapidly declined over the first couple of hours after which it gradually increased again (**Figure 1A**, gray bars), likely corresponding to passing the exponential and entering the stationary phase of growth. Oddly, however, in contrast to its sustaining effect on heat resistance (**Figure 2A**, white bars), the presence of chloramphenicol could not prevent this initial decline of HHP resistance in the unstressed control cells (**Figure 2A**, gray bars). Moreover, in contrast to the slightly increasing heat resistance displayed by the heat stressed survivors upon further incubation (**Figure 1B**, white bars), their massively increased initial HHP resistance progressively declined over the next couple of hours even though the majority of heat stressed survivors did not commit to growth (**Figure 1B**, gray bars). Importantly, the presence of chloramphenicol did not significantly affect the emergence nor evolution of this HHP resistance (**Figure 2B**, gray bars), suggesting that the observed HHP resistance was not caused by a cellular response following the initial heat shock.

## Discussion

Although (mild) thermal processes are commonly used in food preservation, and often serve as part of a hurdle approach, little is known on the resistance behavior of those foodborne bacteria that survive an inimical heat shock or the population that emerges from these survivors. Upon scrutinizing the conduct of heat stressed survivors of *E. coli* O157:H7 ATCC 43888 a number of unanticipated findings emerged. Perhaps most importantly, it was observed that these severely stressed survivors are not by default hypersensitive to a subsequent stress. Indeed, while our data indicate that virtually all of the heat stressed survivors were initially sublethally injured, they were only marginally more sensitive to a subsequent heat treatment and at the same time much more resistant to a subsequent HHP shock in comparison with control cells. Nevertheless, the heat stressed survivors were not composed of mutants displaying a stably increased heat or HHP resistance, underscoring their resistance phenotype to be transient and to stem from stress exposure rather than from genetic changes. Genetically resistant mutants, however, were clearly shown to surface in populations of *Listeria monocytogenes* LO28 exposed to a single HHP treatment (350 MPa, 20 min), where they could make up 25% of the surviving cells. In fact, it could be shown that these mutants spontaneously emerged (and thus pre-existed) in the originally wild-type population and became enriched in the fraction of cells surviving HHP shock (Van Boeijen et al., 2008).

Interestingly, the massive initial HHP resistance displayed by the severely heat shocked survivors seemed not to depend on a translational response, which could suggest that these survivors constitute a phenotypically resistant subfraction that already pre-existed in the original population. However, the progressive fading of this increased HHP resistance and lack of corresponding heat resistance of the same population would argue against this hypothesis. Indeed, if the first (56°C) heat shock would uncover a phenotypically resistant subfraction, these cells would be expected to display increased resistance to the second (53°C) heat shock as well. Therefore, it rather seems that the 56°C heat shock triggered a translation independent response or conferred a peculiar physiological state upon the survivors that somehow mitigated or attenuated the impact of HHP. This state, however, appeared to be transient and the subsequent rapid loss of this peculiar HHP resistance could not even be delayed in the presence of chloramphenicol, indicating that the fading of this state inevitably proceeds despite the block in translation. In the same context, it is interesting to note that while the presence of chloramphenicol could prevent unstressed control cells to enter the exponential phase of growth and thereby sustain their stationary phase heat resistance, it could at the same time not sustain their stationary phase HHP resistance, suggesting that even stationary phase HHP resistance is (in part) supported by a state that cannot be extended by preventing initiation of the exponential growth phase. Aside highlighting the peculiar initial state of severely heat stressed survivors, these observations also reveal fundamental differences in the cellular impact of heat and HHP stress.

The data concerning the differential ampicillin resistance of the heat stressed survivors furthermore suggest that the population emerging from such stressed cells tends to become a mix of (i) non-growing (and perhaps still resuscitating) cells that eventually become diluted by (ii) growing cells that likely mainly originate from early resuscitators. This is in line with previous observations based on the microscopic monitoring of mildly heat or HHP stressed cells that underscore a marked heterogeneity in individual resuscitation times (Govers et al., 2014, Govers et al., 2016; Govers and Aertsen, 2015). As a result, the phenotypic behavior of populations emerging from stressed cells is bound to be less uniform compared to that of isogenic populations stemming from unstressed cells. In fact, in terms of heat resistance it can be anticipated that the non-growing subfraction likely retains a higher resistance than the fraction engaged in exponential growth, although it is unclear whether this resistance is or can be affected by a response. Indeed, while the heat resistance of the heat shocked survivors displays a noticeable increase ca. 4 h after the initial 56°C heat shock, it is difficult to anticipate at what time during resuscitation and/or outgrowth sublethally injured cells would actually be able to mount a (cross)resistance conferring stress response. Indeed, while the heat shock response is well-studied and documented in mildly heat-shocked and therefore more uniformly behaving *E. coli* populations (Chung et al., 2006), it unfortunately remains cryptic whether and how the dynamics of this response can be extrapolated to the small number of cells within a population that manage to survive a severe heat shock. In fact, this ties in with the more general deficit in our understanding of microbial non-growth states (Bergkessel et al., 2016), and it will be interesting to see how the physiology of cells in the persister or the viable-but-non-culturable state would differ from that of sublethally injured resuscitating cells.

In summary, our results reveal the peculiar phenotypic conduct and heterogeneity emerging from a small fraction of sublethally heat-injured survivors of *E. coli* O157:H7, and contribute to a better understanding and anticipation of the dynamics occurring within severely stressed populations of foodborne pathogens, which is becoming indispensable in the context of minimally processed foods. Future work will need to address the molecular mechanisms underlying the observed effects, and how these eventually translate into the behavior of stressed populations in actual food matrices.

## Author Contributions

EG and AA conceived and designed the experiments. EG carried out the experimental work. EG, SG, CM, and AA analyzed the data. EG and AA wrote the manuscript.

## Conflict of Interest Statement

The authors declare that the research was conducted in the absence of any commercial or financial relationships that could be construed as a potential conflict of interest.
